# Numerical Study of Heat Transfer Enhancement within Confined Shell and Tube Latent Heat Thermal Storage Microsystem Using Hexagonal PCMs

**DOI:** 10.3390/mi13071062

**Published:** 2022-06-30

**Authors:** Apichit Maneengam, Sameh E. Ahmed, Abdulkafi Mohammed Saeed, Aissa Abderrahmane, Obai Younis, Kamel Guedri, Muflih Alhazmi, Wajaree Weera

**Affiliations:** 1 Department of Mechanical Engineering Technology, College of Industrial Technology, King Mongkut’s University of Technology North Bangkok, Bangkok 10800, Thailand; apichit.m@cit.kmutnb.ac.th; 2 Department of Mathematics, Faculty of Science, King Khalid University, Abha 62529, Saudi Arabia; sehassan@kku.edu.sa; 3 Department of Mathematics, College of Science, Qassim University, Buraydah 51452, Saudi Arabia; abdulkafi.ahmed@qu.edu.sa; 4 Department of Mathematics, College of Education, Hodeidah University, P.O. Box 3114, Al-Hudaydah 207416, Yemen; 5 Laboratoire de Physique Quantique de la Matière et Modélisation Mathématique (LPQ3M), University of Mascara, Mascara 29000, Algeria; a.aissa@univ-mascara.dz; 6 Department of Mechanical Engineering, College of Engineering at Wadi Addwaser, Prince Sattam Bin Abdulaziz University, Al-Kharj 11991, Saudi Arabia; oubeytaha@hotmail.com; 7 Mechanical Engineering Department, College of Engineering and Islamic Architecture, Umm Al-Qura University, Makkah 21955, Saudi Arabia; kmguedri@uqu.edu.sa; 8 Mathematics Department, Faculty of Science, Northern Border University, Arar 73222, Saudi Arabia; muflih.alhazmi@nbu.edu.sa; 9 Department of Mathematics, Faculty of Science, Khon Kaen University, Khon Kaen 40002, Thailand

**Keywords:** PCM, FEM, tubes, wings, latent heat

## Abstract

Thermophoresis represents one of the most common methods of directing micromachines. Enhancement of heat transfer rates are of economic interest for micromachine operation. This study aims to examine the heat transfer enhancement within the shell and tube latent heat thermal storage system (LHTSS) using PCMs (Phase Change Materials). The enthalpy–porosity approach is applied to formulate the melting situation and various shapes of inner heated fins are considered. The solution methodology is based on the Galerkin finite element analyses and wide ranges of the nanoparticle volume fraction are assumed, i.e., (0% ≤ φ ≤ 6%). The system entropy and the optimization of irreversibility are analyzed using the second law of the thermodynamics. The key outcomes revealed that the flow features, hexagonal entropy, and melting rate might be adjusted by varying the number of heated fins. Additionally, in case 4 where eight heated fins are considered, the highest results for the average liquid percentage are obtained.

## 1. Introduction

Global economic development is rising at a rapid rate, generating an urgent demand for a secure energy source. For a long time, fossil fuels produced energy that met and sustained human needs, and currently account for 87 percent of our energy supply [1]. However, fossil fuel sources are non-renewable, unsustainable, and volatile in price. Additionally, they wreak havoc on the ecosystem and contribute significantly to global warming [2]. These serious concerns have pushed scientists and engineers around the globe to develop solutions for renewable energy production. Due to its great adaptation in enhancing renewable energy production, the PCM-based Thermal Energy Storage (TES) systems are recognized as critical treatment. With the aid of a PCM-based TES system, solar thermal energy may be stored during peak solar hours and subsequently utilized during off-peak hours [3,4,5]. Storage of unused thermal energy might assist in narrowing the gap between energy demand and renewable energy supply [6]. Jesumathy et al. [7] carried out comprehensive research employing paraffin wax as a PCM in horizontal double-tube latent heat thermal energy storage (LHTES) systems.

Their experimental data indicated that conduction and convection were the primary causes of heat transport throughout melting (charging) and solidification (discharging) situations. Zivkovic et al. [8] performed a theoretical investigation of the charging rate of PCMs in rectangular and cylindrical compartments with a horizontal axis. They discovered that in the case of equivalent volume and heated surface area, PCM in a rectangular structure melts quicker than PCM in a cylindrical container. Saeed et al. [9] studied the thermal performance of a new kind of plate thermal energy storage tank in an experimental setting. Their findings indicated that the substantially enhanced design of the storage tank was roughly 83.1 percent effective, despite the limited heat conductivity of the PCM utilized. Tabassum et al. [10] investigated the effect of the inner tube’s cross-sectional form and vertical location within an inverse triangular annulus filled with PCM on the PCM’s melting rate. According to their findings, the heat exchanger with an eccentric inner circular tube positioned vertically gives the greatest energy storage capacity. Pahamli et al. [11] quantitatively studied the influence of eccentricity, inflow temperature, and HTF flow rate on the charging process of RT50 as a PCM installed in the annulus space of a horizontal double-pipe LHTES. The results from this study showed that the downward movement of the inner pipe significantly enhanced the PCM melting process, evidenced by a reduction in the overall melting time by roughly 64 percent. Vyshak et al. [12] conducted a numerical analysis to determine the PCM melting rate in three different enclosures (rectangular, cylindrical, shell and tube). They observed that the shell-and-tube design responds the quickest to melting processes for the same amount of energy provided. Pourakabar et al. [13] conducted research on the melting and solidification of PCM within cylindrical containers with varying shell forms and inner tube layouts. The results indicated that the case with two vertical tubes and the case with a single tube had the greatest and lowest charging rates, respectively. Senapati et al. [14] addressed the eccentricity effects in an annular cylinder with fins statistically. They concluded that there was no discernible difference in natural convection heat transfer rate between a horizontal cylinder with eccentric and concentric fins. Sadeghi et al. [15] investigated the effects of charging and discharging multi-layer PCMs in coaxial cylinders. According to the data, the system can save just 23.28 percent of the intake energy when using a single layer of RT65. While the amount of energy saved inside three-layer PCMs varies according to their thickness and configuration, the total amount of energy saved within the cascaded PCMs is 41.67 percent. Ardahaie et al. [16] conducted a numerical analysis of the charging phase inside an LHTES unit with a flat spiral tube. The research examined a variety of operating scenarios and geometrical design characteristics. Their findings indicated that the melting process of the PCM was substantially affected by the random distribution of the flat spiral tube plane inside the shell. Sodhi et al. [17] numerically evaluated the thermal performance of an LHTES unit with a horizontally conical shell and a coiled tube filled with sodium nitrate as the PCM. Their research focused on optimizing the design of the conical shell. Their findings indicated that the optimal storage values were 98.6 mm and 54 mm for the conical shell’s inlet and outflow diameters, respectively. Shahsavar et al. [18] conducted a numerical study to determine the effect of porous media and surface waviness on the melting and solidification of composite PCM in a vertical double-pipe LHTES. Their findings suggested that when porous structures with high conductivity and wavy channels are used, the overall melting and solidification times may be greatly lowered by up to 91.4 percent and 96.7 percent, respectively.

While a literature review demonstrates that PCM has several disadvantages, including phase separation, corrosion potential, leakage issues, supercooling, poor specific heat, and thermal conductivity, there are solutions available to alleviate or mitigate these disadvantages [19,20,21,22,23,24]. Using performance improvement approaches, the PCM’s phase transition rate, thermal conductivity, latent heat storage capacity, and thermo-physical stability are all enhanced. On the other hand, nanofluids are well known for their ability to enhance heat transfer rates [25,26,27,28]. However, unlike PCM, nanofluids are not able to restore or release heat. Recent studies have found that the phase change rate may be improved with the addition of nanoparticles [29]. This implies a decrease in the time required to store/release thermal energy. In a cylindrical TES unit, Ebadi et al. [30] investigated the effect of nanoparticles on a bio-based nano PCM. They discovered that whereas the early stages of the charging process were the same with or without nanoparticles, the nanoparticles accelerated the phase transition process relative to pure PCM. Sarrafha et al. [31] investigated the thermal transient behavior of MWCNT/PCM implanted in a multi-layered wall. The collected findings indicate that enhancing the thermal conductivity of the PCM by MWCNT inclusion results in a more comfortable thermal environment on chosen winter and fall days but not on summer days. Kashani et al. [32] discussed the effect of surface waviness nanoparticle volume fraction on solidification process of Cu-water nanofluid considered as nano-enhanced PCM (NEPCM). Zirui et al. [33] investigated the heat storage capacity and heat transmission rates of NEPCM (graphene nanoplatelets/1-tetradecanol) melting in a differentially heated rectangular chamber. They discovered that increasing the concentration of GNP results in a slight reduction of both heat storage and heat transmission rates during melting in all geometrical and thermal settings. This suggests that using NEPCM in such a cavity may be ineffective at increasing heat storage rate due to the dramatic increase in viscosity, which significantly deteriorates free convection heat transfer during melting to outweigh the enhanced heat conduction provided by a modest increase in thermal conductivity. Jesumathy et al. [34] investigated the thermal properties of paraffin wax incorporated with nanoparticles of copper oxide. The findings clearly indicated that a higher mass fraction of copper nanoparticles increased the thermal conductivity of the NEPCM. Furthermore, various fin shapes and designs can be employed to enhance PCM/TES thermal performance [35,36,37,38,39]. Abdulateef et al. [40] found that the heat enhancement factor was directly proportional to the number and size of these fins. Duan et al. [41] evaluated the influence of fins on phase change enhancement. The findings suggest that when the fin number is set, PCM’s total melting and solidification times in an annulus with spiral fins may be reduced by up to 57.60 and 74.13 percent, respectively. Sciacovelli et al. [42] investigated the impact of Y-shaped fins inserted in a shell-and-tube LHTES system using numerical simulations. They determined that the unit’s efficiency increased by 24 percent. They note that a large angle between the fins’ branches increases the rate of phase shift. Al-Abidi et al. [43] investigated the PCM charging duration in a triplex finned-tube heat exchanger using numerical simulations. They discovered that the thickness of the fins had a negligible effect on the phase transition rate in comparison to the size and number of the fins. Mazhar et al. [44] radially placed rectangular copper fins around heat exchanger pipes to improve heat transmission in a PCM used for low-grade heat harvesting. Li-Wu Fan et al. [45] investigated the impact of melting temperature and the presence of internal fins on the performance of a PCM-based heat sink used to control the thermal performance of commercial CPUs used in modern personal computers.

In all the aforementioned studies, the authors neglected the case of nano-enhanced phase change materials (NEPCM) and examination of heat transfer enhancement using multi-heated fins. Therefore, this study aims to cover this gap using either the novel shape of LHTES or novel type of the mixture, namely NEPCM. In fact, thermophoresis, which can be defined, as using temperature difference to control movement, is one of the methods used in operating micromachines. Enhancing the heat transfer rate would bring added economical value in directing micromachines. This study’s specific objective is to enhance the design and analysis of LHTES systems for real-world applications. The literature shows that attempts were presented to improve the thermal performance of shell-and-tube LHTES units by augmenting the PCM’s thermal conductivity in the LHTES, and optimizing the design of the LHTES, thereby reducing the required time for charging/discharging the latent energy stored in PCM. The novel design proposes to develop an efficient PCM melting situation by adopting a hexagonal shape for the shell and attaching fins to the tube. Another goal is comparing the performance of NEPCM with improved thermal conductivity with that of pure PCM.

Another important objective of this study is examination of the entropy of the system. In fact, entropy plays a large role in the second law of thermodynamics, which states that atoms tend to become freer and randomly arranged, so that throughout the universe, the level of entropy is rising at a steady rate. With this law also comes the notion that thermal energy moves along a thermal gradient from hotter to colder areas, but does not flow in the opposite direction (from cold to hot). Here, both the entropy due to the heat transfer and entropy due to the fluid friction together with values of the Bejan number are computed and discussed.

## 2. Methodology and Problem Definition

**Figure 1:**
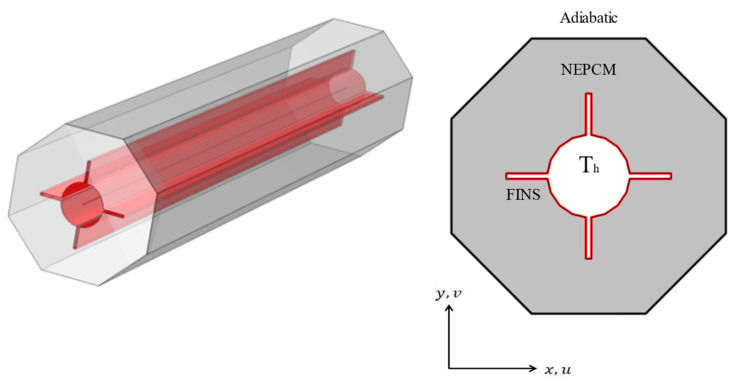
Two-dimensional view of LTESS (right) and 3D view (left).

**Figure 2:**
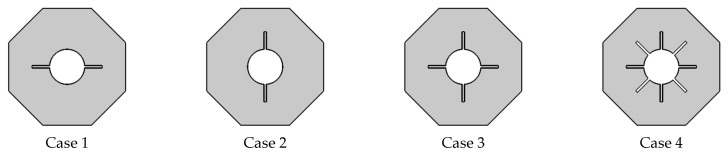
Different cases in current article.

The present computational model is shown in Figure 1. The working fluid is nano-enhanced phase change material (NEPCM) consisting of copper nanoparticles and/ paraffin wax, where Table 1 includes their thermal properties. The effect of fins is investigated with four distinct configurations, as seen in Figure 2. The nano-enhanced PCM begins with a liquidus temperature of 323.15 K. The fins are kept at a high temperature (T_h_ = 333.15 [K]), whilst the hexagonal surfaces are kept adiabatic. The circumradius of the hexagonal shell is L = 21.64 mm, and the radii of the inner circular tubes as well as the fins lengths are 6 mm.

### 2.1. Problem Formulation

We assumed Newtonian and laminar flow to represent this transient phenomenon. The Boussinesq estimate was utilized to account for the gravitational force effect. The following are the formulae, which may be found in [46]:

(1) $$\nabla \cdot \vec{V}=0$$

(2) $$\left(\frac{\partial v}{\partial t}+\vec{V}\cdot \nabla v\right)=\mathrm{vC}\frac{{\left(\lambda -1\right)}^{2}}{\varepsilon +\lambda^{3}}+\frac{1}{\rho_{\mathrm{nf}}}\left(-\nabla P+\mu_{\mathrm{nf}}\nabla^{2}v\right)+\frac{1}{\rho_{\mathrm{nf}}}{\left(\rho \beta \right)}_{\mathrm{nf}}g\left(T-T_{\mathrm{ref}}\right)$$

(3) $$\frac{\partial u}{\partial t}+\vec{V}\cdot \nabla u=\mathrm{uC}\frac{{\left(\lambda -1\right)}^{2}}{\varepsilon +\lambda^{3}}+\frac{1}{\rho_{\mathrm{nf}}}\left(-\nabla P+\mu_{\mathrm{nf}}\nabla^{2}u\right)$$

(4) $${\left({\rho C}_{p}\right)}_{\mathrm{nf}}\frac{\partial {\left(\rho L\lambda \right)}_{\mathrm{nf}}}{\partial t}+{\left({\rho C}_{p}\right)}_{\mathrm{nf}}\frac{\partial T}{\partial t}-k_{\mathrm{nf}}\nabla^{2}T=-{\left({\rho C}_{p}\right)}_{\mathrm{nf}}\vec{V}\cdot \nabla T$$

where $C=1\times 10^{5},\;\varepsilon =0.001$.

The NEPCM properties are predicted using the following single-phase situation:

(5) $${\left({\rho C}_{p}\right)}_{f}^{-1}{\left({\rho C}_{p}\right)}_{\mathrm{nf}}=\left(1-\phi \right)+\phi {\left({\rho C}_{p}\right)}_{s}{\left({\rho C}_{p}\right)}_{f}^{-1}$$

(6) $$\rho_{\mathrm{nf}}=\phi \rho_{s}+\rho_{f}\left(1-\phi \right)\;$$

(7) $${\left(\rho \beta \right)}_{\mathrm{nf}}=\phi {\left(\rho \beta \right)}_{s}+\left(1-\phi \right){\left(\rho \beta \right)}_{f}\;$$

(8) $$\;{\left(\rho L\right)}_{f}=\frac{{\left(\rho L\right)}_{\mathrm{nf}}}{\left(1-\phi \right)}\;$$

(9) $$k_{\mathrm{nf}}=\frac{2k_{f}+2\phi \left(k_{s}-k_{f}\right)+k_{p}}{k_{p}-\phi \left(k_{s}-k_{f}\right)+2k_{f}}k_{f}$$

(10) $$\mu_{\mathrm{nf}}=\frac{\mu_{f}}{{\left(1-\phi \right)}^{2.5}}$$

Enthalpy formulated as:

(11) $$h=h_{\mathrm{ref}}+\int_{T_{\mathrm{ret}}}^{T}{\left(C_{p}\right)}_{\mathrm{nf}}\mathrm{dT}$$

(12) $$=\{\begin{array}{ll} 1 & \;\;\;\;T<T_{l}\\ \frac{T-T_{s}}{T_{l}-T_{s}} & T_{s}<T<T_{l},H_{e}=h+\lambda L\\ 0 & \;\;\;\;T<T_{s} \end{array}\;$$

the formula of HT entropy, FF entropy, and total irreversibility are:

(13) $$\begin{array}{cc} S_{\mathrm{gen},T} & =S_{\mathrm{gen},\mathrm{HT}\;}+S_{\mathrm{gen},\;\mathrm{FF}}\;\\ & =\frac{k_{\mathrm{nf}}}{T^{2}}\left[{\left(\frac{\partial T}{\partial x}\right)}^{2}+{\left(\frac{\partial T}{\partial y}\right)}^{2}\right]\\ & \;+\frac{\mu_{\mathrm{nf}}}{T}\left\{2\left[{\left(\frac{\partial u}{\partial x}\right)}^{2}+{\left(\frac{\partial v}{\partial y}\right)}^{2}\right]+{\left(\frac{\partial u}{\partial y}+\frac{\partial v}{\partial x}\right)}^{2}\right\}\; \end{array}$$

The outer conditions imposed to the aforementioned system are no-slip boundary conditions (u=v=T=0) while on the included fins $\left(u=v=0,\;T=T_{h}\right)$.

### 2.2. GFEM Treatments

**Figure 3:**
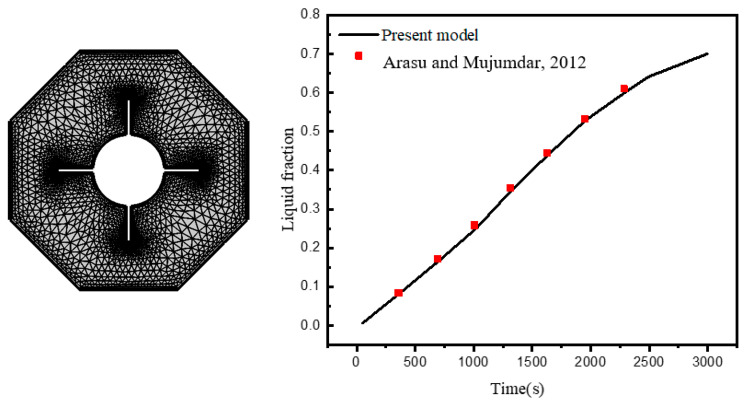
Generation and comparisons of elements with [48].

In the next step, mathematical tools are used to obtain the outcomes. A Galerkin Finite Elements treatment is used to solve the aforementioned PDE’S (1)–(4), which contain the flow and HT processes, together with the imposed B.C’s. The non-strong versions of the governing equations are defined, and a non-uniform grid mesh discretization is applied. The procedure is fully explained in [47]. The current algorithm is validated and shown in Figure 3 utilizing additional numerical data from Arasu and Mujumdar [48]. Based on this number, we may be sure of our conclusions.

## 3. Results and Discussion

The findings of an investigation into the effects of melting on the mixture flow containing a Phase Change Substance (PCS) are presented and analyzed in this section. The working fluid is Cu/paraffin wax in this case and the area of flow is a cylindrical pipe with fins at the cross-section. Isotherms, velocities, and concentration of NP characteristics are investigated for a variety of heating scenarios, including a hexagon with two horizontal fins, a hexagon with two vertical fins, a hexagon with four fins, and a hexagon with eight heated parts. The time interval between shots is between 100 s and 600 s, and the volume percentage of nanoparticles is between 0–0.08. To offer a complete examination of the liquid fraction’s average values, Bejan number Be avg, and HT rate Nu avg are plotted against time over a broad range of the investigated parameters. In addition, the wholly melted condition (liquid fraction = 1) may be utilized to end the calculations.

**Figure 4:**
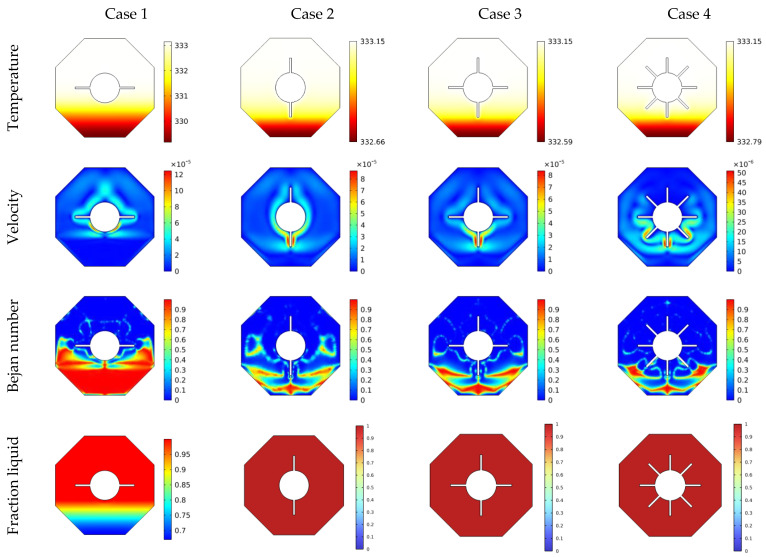
θ(x,y), U(x,y), Be(x,y) and liquid fraction features for various geometries at t = 130 min, φ=0.03.

The isotherms, velocities, local Bejan number, as well as liquid fraction at different situations of inner heated are shown in Figure 4. It is worth observing that the temperature characteristics are gathered close to the fins for all instances, indicating the presence of a non-heated zone around the outer hexagon’s bottom. These temperature distributions peak in C4 (eight fins), indicating a reduction in the aforementioned non-heated area at the lower part. Additionally, it was noted that raising the number of wings reduces the temperature differences, resulting in a decrease in both gradients of the temperature and HT rate. Moreover, as the number of wings increases, a noticeable reduction of velocity values is observed. Physically, raising the number of wings enhances the complexity of the flow region, hence increasing flow resistance. In a related context, the Bejan number characteristics demonstrate that increasing the number of heated fins lowers temperature gradients, and therefore irreversibility of the fluid friction becomes higher. Additionally, the mushy zone is visible at the top half of the area in all considerations, and the heated fins numbers increase the melted region.

**Figure 5:**
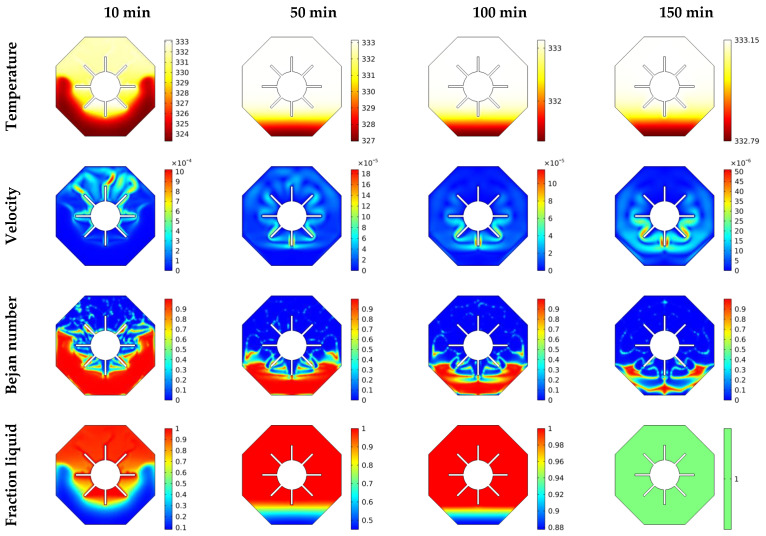
θ(x,y), U(x,y), Be(x,y) and liquid fraction features for various times at C4, φ=0.03.

The isotherms, velocities, local Be issue, and local liquid percentage are all illustrated in Figure 5 as they vary with time. Throughout these calculations, case 3 is employed, which employs an outer hexagon with four fins. Obtained findings revealed that at the start of all computations (short time values), isotherms, velocities, and Bejan number distributions occurred around the included heated region, suggesting the presence of a non-active zone towards the outer limits. As time passes, the suspension begins to convey and disperse the isotherms across the area. Thus, for time = 600, suitable thermal domains are created by increasing the velocity towards the outer boundary’s bottom.

Additionally, the greater time values favor the irreversibility of fluid friction towards the bottom over the irreversibility of heat transport. The physical standpoint states that the mentioned tendency owes to the fact that as time passes, the velocity gradients become more pronounced, causing an irreversible rise in friction of the fluid layers. Additionally, the mushy area is visible throughout the whole flow domain as time values grow.

**Figure 6:**
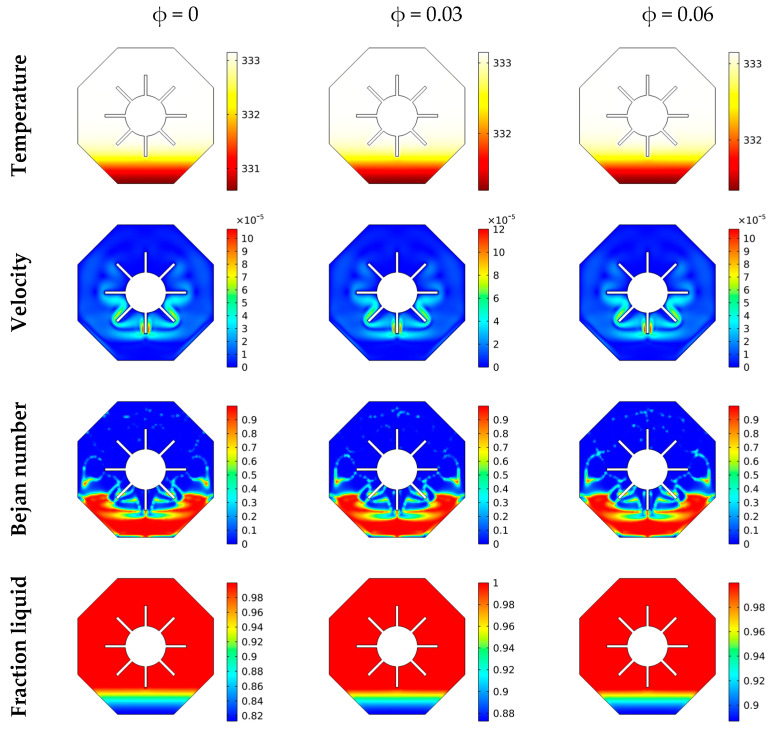
θ(x,y), U(x,y), Be(x,y) and liquid fraction features for various φ at t = 100 min, C4.

The isotherms, velocities, local Bejan number, and liquid fraction distributions are shown in Figure 6 as a function of the NP concentrations. In this situation, outer hexagons, including four fins, are employed. It is noticed that with increases in the mixture’s viscosity, there is a decrease in convective transport. The findings suggest that the velocity and temperature gradients reduce as it grows. Additionally, with low values of φ, the domain Bejan number occurs close to the wings rather than the lower borders. On the contrary, increasing φ  increases the mushy region inside the active flow area until it demonstrates entirely melted behaviors at  φ≥0.06. Major changes in the values of the velocity and melting features can be noted as φ increases. Here, the value φ=0.03 gives a higher velocity and melting process compared to other values of φ.

**Figure 7:**
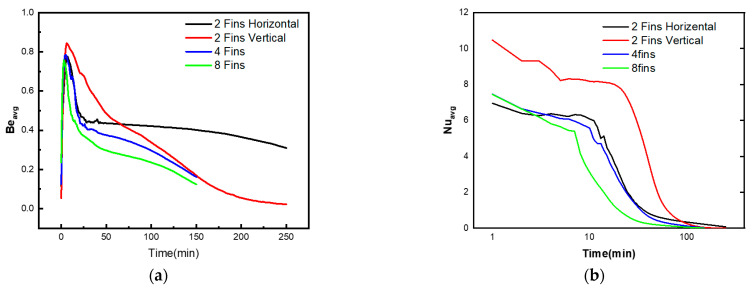

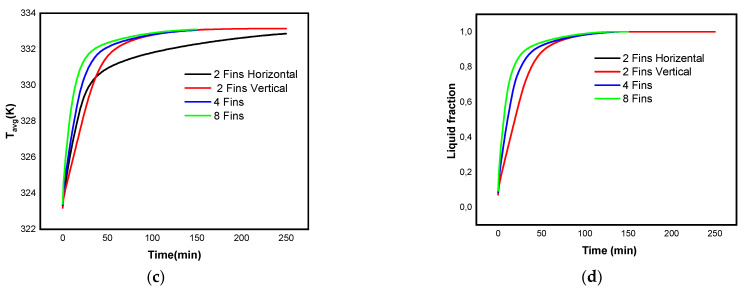
Behaviors of (**a**) ${\mathrm{Be}}_{\mathrm{avg}}$, (**b**) ${\mathrm{Nu}}_{\mathrm{avg}}$, (**c**) $T_{\mathrm{avg}}$, and (**d**) liquid fraction for various cases of the inner fins.

**Figure 8:**
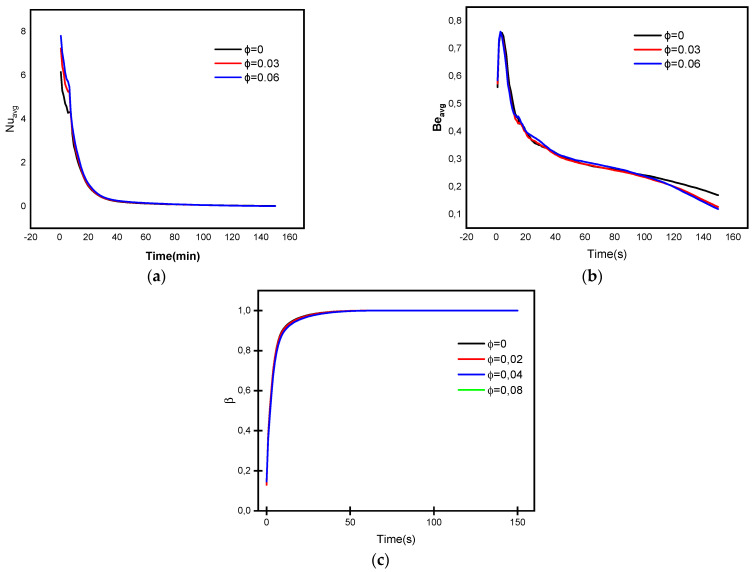
Behaviors of (**a**) ${\mathrm{Nu}}_{\mathrm{avg}}$, (**b**) ${\mathrm{Be}}_{\mathrm{avg}}$, and (**c**) β for various concentrations of NP.

Figure 7 and Figure 8 depict the liquid fraction mean values, means Bejan number Be_avg_, and mean Nusselt coefficient Nu_avg_ profiles for the impact of the heated fins count, time parameter, and NP concentration parameter. The findings indicated to C4, which assumes eight heated fins, resulted in the higher mean liquid fraction behaviors. Moreover, when the issue of heated fins increases, the mean Bejan and Nusselt values decline. In addition, the mean rate of heat transmission decreases as the temperature gradients lessen. Furthermore, the higher values result in a greater dominance of heat transfer irreversibility over fluid friction irreversibility. Finally, it was noted that enhancing the volume fraction parameter increases the mushy zone, resulting in an increase in the average liquid fraction.

## 4. Conclusions

This article provides a numerical analysis of the melting effects on the convection of Phase Change Substance in hexagon-shaped containers with heated cross-sections. Based on the number of heated wings, four instances were considered: C1 (two-horizontal fins), C2 (two-vertical fins), C3 (four fins), and C4 (eight-heated parts). We investigated the buoyancy-driven flow and assumed entirely melted circumstances. The governing system was solved using the Finite Elements Technique (FET), and the pressure was computed using the Poisson formula. Significant discoveries include the following: Temperature, velocity, and Bejan number distributions increase when the heated wings are augmented owing to the buoyancy–convective scenario being enhanced.

Additionally, in C4, the melted region is confined to the majority of the flow domain.

At low time values, isotherms, velocities, and the liquid fraction are seen close to the inner heated parts, while time progression results in the formation of a suitable temperature and melting of flow hexagons.

The rise in NP concentration increases the mixture’s dynamic viscosity, and hence the velocities decrease as φ grows.

As time passes, the entropy caused by viscous dissipation becomes more critical than the irreversibility caused by heat transfer.

The presented results recommend the use of two heated fins in the design of LHTSS to obtain a higher rate of heat transfer.

Using a 6% concentration of NP (nanoparticles) is recommended to reduce the heat transfer irreversibility within LHTSS.

## Figures and Tables

**Table 1:** Summary of the properties of nanoparticles and PCM.

Property	Cu	Paraffin Wax (Liquid/Solid)	
$\rho \left[\mathrm{kg}/m^{3}\right]$	8954	775	833.6
$\beta \times 10^{5}\left[K^{-1}\right]$	1.67	714	
k [w/mK]	400	0.15	0.15
L [KJ/kg]	-	184.48	
Melting temperature (K)	-	54.32	
$\mu \times 10^{3}\;$[Pa.s]	-	6.3	
$C_{p}\;\left[J/\mathrm{kgK}\right]$	383	2440	2384

## Nomenclature

C	Mushy zone morphology constant	
$C_{p}$	Heat capacity (J/kg.K)	
g	Gravity acceleration $(m.s^{-2}$)
$h_{\mathrm{ref}}$	Reference sensible enthalpy	
k	Thermal conductivity (W/m.K)
$L_{\;}$	Latent heat coefficient (KJ/kg)	
p	Pressure ($N/m^{2}$)	
$S_{\mathrm{gen},T}$	Total system entropy	
$S_{\mathrm{gen},\mathrm{HT}}$	Heat tranfer entropy	
$S_{\mathrm{gen},\mathrm{FF}}$	Fluid friction entropy	
t	Time (s)	
T	Temperature (K)	
$T_{s}$	PCM’s solid temperature (K)	
$T_{l}$	PCM’s liquid temperature (K)	
(u,v)	Velocity components (m/s)	
PCM	Phase change material	
2D	Two-dimensional	
3D	Three-dimensional	
LHTES	Latent heat thermal energy storage	
HTF	Heat transfer fluid	
NEPCM	Nanoparticles-enhanced PCM	
FEM	Finite Element Method	
ρ	Density $\left(\mathrm{Kg}/m^{3}\right)$	
μ	Dynamic viscosity (Pa⋅s)	
α	Thermal diffusivity $\left(m^{2}/s\right)$	
β	Thermal expansion coefficient (1/K)	
ϕ	Volume fraction	
ref	Reference case	
nf	Nanofluid	
s	Solid	
f	Fluid	

## References

[1] Saeed A.M., Abderrahmane A., Qasem N.A.A., Mourad A., Alhazmi M., Ahmed S.E., Guedri K. (2022). A numerical investigation of a heat transfer augmentation finned pear-shaped thermal energy storage system with nano-enhanced phase change materials. J. Energy Storage.

[2] Zandalinas S.I., Fritschi F.B., Mittler R. (2021). Global Warming, Climate Change, and Environmental Pollution: Recipe for a Multifactorial Stress Combination Disaster. Trends Plant Sci..

[3] Qasem N.A., Abderrahmane A., Younis O., Guedri K., Said Z., Mourad A. (2022). Effect of a rotating cylinder on convective flow, heat and entropy production of a 3D wavy enclosure filled by a phase change material. Appl. Therm. Eng..

[4] Al-Kouz W., Aissa A., Devi S.S.U., Prakash M., Kolsi L., Moria H., Jamshed W., Younis O. (2022). Effect of a rotating cylinder on the 3D MHD mixed convection in a phase change material filled cubic enclosure. Sustain. Energy Technol. Assess..

[5] Abderrahmane A., Hatami M., Younis O., Mourad A. (2022). Effect of double rotating cylinders on the MHD mixed convection and entropy generation of a 3D cubic enclosure filled by nano-PCM. Eur. Phys. J. Spec. Top..

[6] Yang T., King W.P., Miljkovic N. (2021). Phase change material-based thermal energy storage. Cell Rep. Phys. Sci..

[7] Jesumathy S.P., Udayakumar M., Suresh S., Jegadheeswaran S. (2014). An experimental study on heat transfer characteristics of paraffin wax in horizontal double pipe heat latent heat storage unit. J. Taiwan Inst. Chem. Eng..

[8] Zivkovic B., Fujii I. (2001). An analysis of isothermal phase change of phase change material within rectangular and cylindrical containers. Sol. Energy.

[9] Saeed R.M., Schlegel J.P., Sawafta R., Kalra V. (2019). Plate type heat exchanger for thermal energy storage and load shifting using phase change material. Energy Convers. Manag..

[10] Tabassum T., Hasan M., Begum L. (2017). 2-D numerical investigation of melting of an impure PCM in the arbitrary-shaped annuli. Int. J. Therm. Sci..

[11] Pahamli Y., Hosseini M.J., Ranjbar A.A., Bahrampoury R. (2018). Inner pipe downward movement effect on melting of PCM in a double pipe heat exchanger. Appl. Math. Comput..

[12] Vyshak N.R., Jilani G. (2007). Numerical analysis of latent heat thermal energy storage system. Energy Convers. Manag..

[13] Pourakabar A., Darzi A.A.R. (2019). Enhancement of phase change rate of PCM in cylindrical thermal energy storage. Appl. Therm. Eng..

[14] Senapati J.R., Dash S.K., Roy S. (2016). 3D numerical study of the effect of eccentricity on heat transfer characteristics over horizontal cylinder fitted with annular fins. Int. J. Therm. Sci..

[15] Sadeghi H.M., Babayan M., Chamkha A. (2020). Investigation of using multi-layer PCMs in the tubular heat exchanger with periodic heat transfer boundary condition. Int. J. Heat Mass Transf..

[16] Ardahaie S.S., Hosseini M.J., Ranjbar A.A., Rahimi M. (2019). Energy storage in latent heat storage of a solar thermal system using a novel flat spiral tube heat exchanger. Appl. Therm. Eng..

[17] Sodhi G.S., Jaiswal A.K., Vigneshwaran K., Muthukumar P. (2019). Investigation of charging and discharging char-acteristics of a horizontal conical shell and tube latent thermal energy storage device. Energy Convers. Manag..

[18] Shahsavar A., Al-Rashed A.A.A.A., Entezari S., Sardari P.T. (2019). Melting and solidification characteristics of a double-pipe latent heat storage system with sinusoidal wavy channels embedded in a porous medium. Energy.

[19] Rostami S., Afrand M., Shahsavar A., Sheikholeslami M., Kalbasi R., Aghakhani S., Shadloo M.S., Oztop H.F. (2020). A review of melting and freezing processes of PCM/nano-PCM and their application in energy storage. Energy.

[20] Magendran S.S., Khan F.S.A., Mubarak N., Vaka M., Walvekar R., Khalid M., Abdullah E., Nizamuddin S., Karri R.R. (2019). Synthesis of organic phase change materials (PCM) for energy storage applications: A review. Nano Struct. Nano Objects.

[21] Waqas A., Ji J., Xu L., Ali M., Zeashan, Alvi J. (2018). Thermal and electrical management of photovoltaic panels using phase change materials—A review. Renew. Sustain. Energy Rev..

[22] Gholamibozanjani G., Farid M. (2021). A Critical Review on the Control Strategies Applied to PCM-Enhanced Buildings. Energies.

[23] Wu S., Yan T., Kuai Z., Pan W. (2020). Thermal conductivity enhancement on phase change materials for thermal energy storage: A review. Energy Storage Mater..

[24] Leong K.Y., Abdul Rahman M.R., Gurunathan B.A. (2019). Nano-enhanced phase change materials: A review of thermo-physical properties, applications and challenges. J. Energy Storage.

[25] Alharbi K.A.M., Ahmed A.E.-S., Sidi M.O., Ahammad N.A., Mohamed A., El-Shorbagy M.A., Bilal M., Marzouki R. (2022). Computational Valuation of Darcy Ternary-Hybrid Nanofluid Flow across an Extending Cylinder with Induction Effects. Micromachines.

[26] Rasool G., Saeed A.M., Lare A.I., Abderrahmane A., Guedri K., Vaidya H., Marzouki R. (2022). Darcy-Forchheimer Flow of Water Conveying Multi-Walled Carbon Nanoparticles through a Vertical Cleveland Z-Staggered Cavity Subject to Entropy Generation. Micromachines.

[27] Chabani I., Mebarek-Oudina F., Ismail A.A.I. (2022). MHD Flow of a Hybrid Nano-Fluid in a Triangular Enclosure with Zigzags and an Elliptic Obstacle. Micromachines.

[28] Khetib Y., Abo-Dief H.M., Alanazi A.K., Sajadi S.M., Sharifpur M., Meyer J.P. (2021). A Computational Fluid Dynamic Study on Efficiency of a Wavy Microchannel/Heat Sink Containing Various Nanoparticles. Micromachines.

[29] Yang L., Huang J.N., Zhou F. (2020). Thermophysical properties and applications of nano-enhanced PCMs: An update review. Energy Convers. Manag..

[30] Ebadi S., Tasnim S.H., Aliabadi A.A., Mahmud S. (2018). Geometry and nanoparticle loading effects on the bio-based nano-PCM filled cylindrical thermal energy storage system. Appl. Therm. Eng..

[31] Sarrafha H., Kasaeian A., Jahangir M.H., Taylor R.A. (2021). Transient thermal response of multi-walled carbon nanotube phase change materials in building walls. Energy.

[32] Kashani S., Ranjbar A.A., Abdollahzadeh M., Sebti S. (2012). Solidification of nano-enhanced phase change material (NEPCM) in a wavy cavity. Heat Mass Transf..

[33] Li Z., Hu N., Tu J., Fan L. (2020). Experimental Investigation of Heat Storage and Heat Transfer Rates during Melting of Nano-Enhanced Phase Change Materials (NePCM) in a Differentially-Heated Rectangular Cavity. J. Therm. Sci..

[34] Jesumathy S., Udayakumar M., Suresh S. (2012). Experimental study of enhanced heat transfer by addition of CuO nanoparticle. Heat Mass Transf. Stoffuebertragung.

[35] Mousavi S., Rismanchi B., Brey S., Aye L. (2021). PCM embedded radiant chilled ceiling: A state-of-the-art review. Renew. Sustain. Energy Rev..

[36] Naghavi M.S., Metselaar H.S.C., Ang B.C., Zamiri G., Esmailzadeh A., Nasiri-Tabrizi B. (2021). A critical assessment on synergistic improvement in PCM based thermal batteries. Renew. Sustain. Energy Rev..

[37] Mourad A., Aissa A., Said Z., Younis O., Iqbal M., Alazzam A. (2022). Recent advances on the applications of phase change materials for solar collectors, practical limitations, and challenges: A critical review. J. Energy Storage.

[38] Ahmed S.E., Abderrahmane A., Alotaibi S., Younis O., Almasri R.A., Hussam W.K. (2021). Enhanced Heat Transfer for NePCM-Melting-Based Thermal Energy of Finned Heat Pipe. Nanomaterials.

[39] Ashouri M., Hakkaki-Fard A. (2021). Improving the performance of the finned absorber inclined rooftop solar chimney combined with composite PCM and PV module. Sol. Energy.

[40] Abdulateef A.M., Mat S., Abdulateef J., Sopian K., Al-Abidi A.A. (2018). Geometric and design parameters of fins employed for enhancing thermal energy storage systems: A review. Renew. Sustain. Energy Rev..

[41] Duan J., Xiong Y., Yang D. (2020). Study on the effect of multiple spiral fins for improved phase change process. Appl. Therm. Eng..

[42] Sciacovelli A., Gagliardi F., Verda V. (2015). Maximization of performance of a PCM latent heat storage system with innovative fins. Appl. Energy.

[43] Al-Abidi A.A., Mat S., Sopian K., Sulaiman M.Y., Mohammad A.T. (2013). Internal and external fin heat transfer enhancement technique for latent heat thermal energy storage in triplex tube heat exchangers. Appl. Therm. Eng..

[44] Mazhar A.R., Shukla A., Liu S. (2020). Numerical analysis of rectangular fins in a PCM for low-grade heat harnessing. Int. J. Therm. Sci..

[45] Fan L.W., Xiao Y.Q., Zeng Y., Fang X., Wang X., Xu X., Yu Z.T., Hong R.H., Hu Y.C., Cen K.F. (2013). Effects of melting temperature and the presence of internal fins on the performance of a phase change material (PCM)-based heat sink. Int. J. Therm. Sci..

[46] Sheikholeslami M. (2018). Solidification of NEPCM under the effect of magnetic field in a porous thermal energy storage enclosure using CuO nanoparticles. J. Mol. Liq..

[47] Al-Kouz W., Bendrer B.A.I., Aissa A., Almuhtady A., Jamshed W., Nisar K.S., Mourad A., Alshehri N.A., Zakarya M. (2021). Galerkin finite element analysis of magneto two-phase nanofluid flowing in double wavy enclosure comprehending an adiabatic rotating cylinder. Sci. Rep..

[48] Arasu A.V., Mujumdar A.S. (2012). Numerical study on melting of paraffin wax with Al_2_O_3_ in a square enclosure. Int. Commun. Heat Mass Transf..

